# Modelling the behaviour of thermal energy harvesting devices with phase-change materials

**DOI:** 10.1038/s41598-021-00079-y

**Published:** 2021-10-15

**Authors:** Vladimir Kulish, Tomáš Hyhlík, Pavel Sláma

**Affiliations:** Department of Thermodynamics and Fluid Mechanics, Faculty of Mechanical Engineering, Czech Technical University in Prague, Prague, Czech Republic

**Keywords:** Thermodynamics, Mechanical engineering

## Abstract

This paper presents a new general theoretical model of thermal energy harvesting devices (TEHDs), which utilise phase-change materials (PCMs) for energy storage. The model's major goal is to identify a set of parameters under which these devices perform optimally, that is, attain the largest thermal buffering capacity and exchange heat with the surrounding phase as quickly as possible. For the first time, an expression for the characteristic harvesting time is developed from the constructal theory viewpoint under the optimal performance assumption, and a dimensionless criterion that characterizes PCM performance is provided. Furthermore, a new non-field solution of the energy equation governing the process of heat transfer within TEHDs with PCMs has also been derived. An expression for the effective thermal effusivity is then obtained. Finally, under a given set of boundary conditions and geometrical constraints, a novel simple technique for the optimal choice of PCMs in TEHDs has been established.

## Introduction

Sparing energy, more efficient transport and conservation of energy, as well as more efficient cooling of systems generating heat during their operation are among the most important tasks to be tackled by modern science and technology.

As pointed out in the authors’ previous work, phase-change materials (PCMs) are gaining a lot of attention as a sustainable approach to store energy at high ambient temperatures and release it at lower ones, thus buffering undesirable temperature oscillations^1^. Among the recent works, two excellent papers by Lin and coauthors provide the most comprehensive reviews on the state of the art in PCM applications and modelling^2,3^. As can be seen from these reviews, in spite of significant growth of the body of knowledge about PCM performance and applications, some lack of understanding is still present. In particular, no quantitative criteria are known for choosing PCM physical properties to provide their optimal performance.

Diurnal temperature differences, peak power usage of electronic components, and solar heat exposure are some examples of transient energy sources that should be dissipated as rapidly as possible, or even better, saved for use when the temperature drops. These applications dictate the choice of the *desired properties of PCMs,* such as their *high thermal buffering capacity* and *ability to exchange heat quickly with adjacent (matrix) materials*. In view of the fact that the thermal buffering capacity cannot be represented by a single quantity, because it is defined by several integral parameters (e.g., latent heat and dimensions of the PCMs), the ability to exchange heat with a matrix material is best described by the thermal effusivity otherwise known as thermal inertia or thermal permeability^4^.

This paper presents a theoretical model of the behaviour of thermal energy harvesting devices (TEHDs), in which PCMs are used for energy storage. The main aim of the model is to establish a set of conditions, under which TEHDs with PCMs operate in an optimal way, that is, achieve the highest thermal buffering capacity and, at the same time, rapidly exchange heat with the adjacent phase (matrix material).

The study presented here originates from a practical need to develop a model of the TEHDs with PCMs in the course of designing a 300 kW heat accumulator for district heating networks. The accumulator is intended to be connected with the secondary system of the heat transfer station. The output medium from such an accumulator is heated water intended for supplying the house heating. The accumulator uses PCM substances to accumulate heat. This heat is transferred to the heat transfer medium by both convection and conduction through a designed metal interface. The input heat transfer medium to the accumulator is the returning water, which has fully transferred its heat within the transfer stations of individual houses. The purpose of heat accumulation in district heating systems is to significantly reduce the load on the heat source and distribution network at the time of maximum consumption and in an effort to use the production and transmission capacity of the entire system at the time of minimum consumption. The result is a significant reduction in the size of these heat sources and networks (e.g. boilers, valves, pipes, etc.), as well as a reduction in heat and hydraulic losses of these production and distribution systems.

The starting point of this study is the constructal theory^5,6^, which defines the frame for establishing a set of criteria for optimal performance of TEHDs with PCMs. In the next section, an expression for the characteristic harvesting time is derived under the optimal performance assumption from the constructal theory viewpoint.

“Problem formulation” of this paper presents the general theoretical formulation of the problem in question. In this section, for the first time. An expression to quantify the volumetric heat sink associated with the presence of the PCM is derived there as well.

In the beginning of “Solution procedure“, for the first time, a dimensionless criterion, which characterises the PCM performance is introduced. Further in the same section, a new non-field solution of the energy equation governing the heat transfer process within TEHDs with PCMs has been obtained by the method of Kulish^7–11^. By this the method has been extended to solving optimization problems. From the solution thus obtained it follows that the thermal effusivity of the matrix material is one of the two parameters, which control the performance of TEHDs with PCMs. An expression for the effective thermal effusivity is then derived, which combines both the thermal effusivity of the matrix material and the dimensionless criterion of the PCM performance introduced earlier.

“Some practically important cases of the boundary conditions” presents the analysis of several important cases of the boundary conditions imposed on TEHDs with PCMs. In particular, the case of periodic or quasi-periodic boundary conditions is considered in more detail, because this case is important if energy harvesting is to be accomplished during diurnal cycles.

From the expression of the dimensionless criterion, which characterises the PCM performance, it follows that the volume fraction of PCMs plays an important role in the energy harvesting process. Hence, “Geometrical constraints on the PCM” is devoted to establishing an optimal value of the volume fraction of PCMs within the matrix material. In the same section, for the first time, the issue of the PCM particles distribution within the matrix material is discussed as well as the geometrical constraints related to the size of those particles. In addition, it has been shown how the thermal diffusing capacity of TEHDs with PCMs is related to the effective diffusivity of the constituent materials involved in TEHDs design.

The last section of the paper provides the general discussion of the model developed in this study. The main aim of this section is to formulate a new concise procedure for the best choice of PCMs in TEHDs under a given set of the boundary conditions and geometrical constraints.

## Optimal performance of thermal energy harvesting devices

In this study, criteria for optimal performance of thermal energy harvesting devices will be derived from the constructal law.

The constructal law is the law of physics that accounts for the phenomenon of evolution (configuration, form, design) throughout nature, inanimate flow systems and animate systems together. In its present form, the law was stated by Bejan in 1997 as follows^5,6^:

*For a finite-size system to persist in time (to live), it must evolve in such a way that it provides easier access to the imposed currents that flow through it.*

Reformulated for the case of thermal energy harvesting devices, the constructal law states that, the optimal performance of these devices is achieved when a given amount of thermal energy is stored within the shortest time span possible. In practice, this means maximising the energy storage rate (power) consumed by the PCMs.

Thus, for a given amount of thermal energy to be harvested, ${Q}_{hv}$, the optimal performance of the energy harvesting device is achieved if

1 $${\stackrel{ }{Q}}_{hv,max}=\frac{{Q}_{hv,max}}{{t}_{hv,min}},$$

where ${t}_{hv,min}$ is the characteristic harvesting time.

The amount of thermal energy, which can be harvested, is given by a simple relation, namely:

2 $${Q}_{hv}={m}_{PCM}{h}_{f}$$

where ${m}_{PCM}$ is the total mass of the PCM used for the harvesting purpose and ${h}_{f}$ is the PCM enthalpy of fusion.

From the latter equation, it may look like the task of maximising the amount of thermal energy harvested reduces to a trivial increase of the PCM total mass and choosing the PCM with the highest possible value of the enthalpy of fusion. It will be shown here, however, that geometric restrictions, imposed on the heat transfer process, define a constraint on the size of PCM particles and their relative position with respect to each other. This, in turn, sets the constraint on the total mass of PCM. Furthermore, the characteristic harvesting time is also related to the total mass of PCM.

Indeed, for a thermal energy harvesting device to perform in an optimal way, its operation cycle should exclude any idle periods. In other words, the amount of the thermal energy harvested must equal the amount of heat delivered to the PCM through the matrix material by conduction (thermal diffusion), that is,

3 $${Q}_{hv}={Q}_{cond}$$

The latter expression is, of course, nothing else but the conservation of energy principle applied to the case in question.

The amount of heat delivered by thermal diffusion is given by

4 $$Q={q}^{{{\prime\prime}}}{A}_{PCM}{t}_{hv},$$

where ${q}^{{{\prime\prime}}}$ is the heat flux through the surface of the PCM particle and ${A}_{PCM}$ is the total surface area of PCM available for energy harvesting.

The heat flux in the latter equation can be estimated as

5 $${q}^{{{\prime\prime}}}=k\frac{\Delta T}{\ell },$$

where $k$ is the thermal conductivity of the matrix material, $\Delta T$ is the characteristic temperature difference, and $\ell$ denotes the average distance between the PCM particles within the matrix material. The characteristic temperature difference is the difference between the phase-change (e.g., melting) temperature and the ambient temperature.

For Eq. (3) to remain valid during the entire operation cycle, the average distance between the PCM particles within the matrix material must be as close as possible to the characteristic length of thermal diffusion, that is,

6 $$\ell =\sqrt{\alpha {t}_{hv}},$$

where $\alpha =k/(\rho {c}_{p})$ is the thermal diffusivity of the matrix material of the density $\rho$ and specific heat capacity ${c}_{p}$, respectively.

From combining Eqs. (2)–(6) and after some algebra follows the expression for the characteristic harvesting time

7 $${t}_{hv}={\left(\frac{{m}_{PCM}{h}_{f}}{\epsilon {A}_{PCM}\Delta T}\right)}^{2},$$

where

8 $$\epsilon =\sqrt{k\rho {c}_{p}}$$

is known as the thermal effisivity^4^, the significance of which will be discussed in the end of this section.

As can be easily seen from the latter equation, the characteristic harvesting time in general depends on the ratio of physical properties of the PCM and matrix material as well as the size of the PCM particles. It is also worth noting that the characteristic temperature difference, $\Delta T$, plays a crucial role as well. This temperature difference is defined from the boundary conditions imposed on the thermal energy harvesting device and the phase-change temperature of the PCM.

Observe that the result given by Eq. (7) is, in its mathematical form, practically identical to the result reported by Bejan^12^. Hence, the model developed in this study can, in principle, be easily extended to thermal energy harvesting devices operating in a periodic (“pulsating”) mode.

As will be shown in the following sections, the thermal effusivity plays an important role in defining the performance of TEHDs. In fact, it is one of the two most important parameters, which control the behaviour of TEHDs. Hence, the physical meaning of the thermal effusivity is discussed here in more detail.

The thermal effusivity of a material is a measure of its ability to exchange thermal energy with its surroundings. The effusivity (thermal inertia) is defined as the square root of the product of the material's thermal conductivity and its volumetric heat capacity (see Eq. 8).

From Eq. (8), it is easy to notice that the units, in which the effusivity is measured, include s^−1/2^. Hence, if the effusivity is to be a parameter in a certain equation, only its product with the square root of time may lead to physically meaningful quantities. This feature is further explored in the following sections.

Furthermore, it is also worth noticing here that, if two semi-infinite bodies initially at temperatures *T*_1_ and *T*_2_ are brought in perfect thermal contact, the temperature at the contact surface will be given by their relative effusivities:

9 $${T}_{m}={T}_{1}+({T}_{2}-{T}_{1})\frac{{\epsilon }_{2}}{{\epsilon }_{2}+{\epsilon }_{1}}$$

Equation (9) is valid for all times for semi-infinite bodies in perfect thermal contact. It is also a good first guess for the initial contact temperature for finite bodies.

From the heat transfer viewpoint, a finite-size body can be treated as semi-infinite (thermally thick) as long as its characteristic length scale, $\ell$, remains less than or equal to the corresponding length scale of thermal diffusion, ${\ell }_{th}$, that is,

10 $$\ell \le {\ell }_{th}=\sqrt{\alpha t}$$

where α denotes the thermal diffusivity of the material and *t* is time.

Because the thermal diffusivity is $\alpha =k/(\rho {c}_{p})$, the time scale, during which a finite-size body can be treated as semi-infinite is

11 $${t}^{*}\le {\left(\frac{\epsilon \ell }{k}\right)}^{2}$$

Therefore, under the assumptions given by Eqs. (3) and (6), the heat transfer domain in this study can be treated as semi-infinite—at least, as long as the PCMs within a given TEHD operate near their phase-change temperatures.

To conclude this section, it is worth noting that, although Eq. (4) implicitly imposes a certain constraint on the size of PCM particles through their total surface area, the exact size constraint can be defined only after an assumption about the shape of PCM particles. The detailed analysis of this is presented in “Geometrical constraints on the PCM”.

## Problem formulation

The heat transfer domain composed of a matrix material, in which PCM is imbedded, is governed by

12 $$\frac{\partial T(r,t)}{\partial t}=\alpha \frac{{\partial }^{2}T(r,t)}{\partial {r}^{2}}-\frac{1}{\rho {c}_{p}}{\stackrel{ }{q}}^{{{\prime\prime\prime}}}(r,t)$$

where ${\stackrel{ }{q}}^{{{\prime\prime\prime}}}(r,t)$ models the effective volumetric heat sink associated with the presence of the PCM. The minus sign in front of the last term in the right side of Eq. (12) shows that heat is depleted by the PCM.

Note that Eq. (12) was obtained under the assumption that the Fourier law of heat conduction is valid^13^, namely

13 $${q}^{{{\prime\prime}}}\left(r,t\right)=-k\frac{\partial T\left(r,t\right)}{\partial r}$$

that is, no ultra-fast heat transfer processes are involved and, hence, no thermal waves are present^14^.

The domain of interest is initially in the state of thermal equilibrium, that is, $T(r,0)={T}_{0}=const$. The two boundary conditions, which are to be imposed on Eq. (12), are not provided intentionally. This will become clear from the following sections.

### Modelling the volumetric heat sink associated with the presence of the PCM

The total amount of heat, consumed by the PCM, is

14 $${Q}_{PCM}={Q}_{heat}+{Q}_{f}$$

where the amount of energy necessary to heat the PCM from its initial temperature, *T*_0_, to the fusion temperature, ${T}_{f}$, equals

15 $${Q}_{heat}={m}_{PCM}{c}_{p,PCM}({T}_{f}-{T}_{0})$$

whereas the amount of heat necessary to change the PCM phase equals

16 $${Q}_{f}={m}_{PCM}{h}_{f}$$

where *h*_*f*_, is the enthalpy of fusion of the PCM. In Eqs. (15) and (16), ${m}_{PCM}={\rho }_{PCM}{V}_{PCM}$ denotes the total mass of the PCM within the matrix material; ${\rho }_{PCM}$ and ${V}_{PCM}$ are the density of the PCM and its total volume, respectively.

Hence, the strength of the volumetric heat sink, associated with the presence of the PCM, can be quantified as

17 $${q}^{{{\prime\prime\prime}}}=\varphi {\rho }_{PCM}\left[{c}_{p,PCM}\left({T}_{f}-{T}_{0}\right)+{h}_{f}\right]$$

where $\varphi ={V}_{PCM}/V$ denotes the volume fraction of the PCM.

Finally, because the characteristic time of the heat transfer process is ${t}_{hv}={\ell }^{2}/\alpha$ from Eq. (6), the volumetric power of the PCM heat sink is

18 $${\stackrel{ }{q}}^{{{\prime\prime\prime}}}={q}^{{{\prime\prime\prime}}}/{t}_{hv}=\varphi \frac{k}{{\ell }^{2}}\frac{{\rho }_{PCM}}{\rho {c}_{p}}\left[{c}_{p,PCM}\left({T}_{f}-{T}_{0}\right)+{h}_{f}\right]$$

In the following section a non-field solution of Eq. (12) for the boundary values of temperature and heat flux is to be derived^11^.

It is worth noting here that non-field solutions were not used in previous studies for analysis of optimization problems. Hence, the material, presented in the following section, is of methodological importance.

## Solution procedure

Upon introducing the dimensionless independent variables $\xi =r/\ell$ and $\tau =\alpha t/{\ell }^{2}$, the excess temperature $\hat{T}(\xi ,\tau )=T(\xi ,\tau )-{T}_{0}$, and the dimensionless temperature $\theta (\xi ,\tau )=\hat{T}(\xi ,\tau )/{\hat{T}}_{f}$, Eq. (12) becomes

19 $$\frac{\partial \theta }{\partial \tau }=\frac{{\partial }^{2}\theta }{\partial {\xi }^{2}}-K$$

where

20 $$K=\varphi \left[\frac{(\rho {c}_{p}{)}_{PCM}}{\rho {c}_{p}}+\frac{(\rho {h}_{f}{)}_{PCM}}{\rho {c}_{p}{\hat{T}}_{f}}\right]$$

denotes the dimensionless criterion, which characterises the PCM performance.

The initial condition, imposed on Eq. (19), becomes $\theta (\xi ,0)=0$.

Upon applying the Laplace transform with respect to $\tau$ to Eq. (19), the latter becomes

21 $${\Theta }^{{{\prime\prime}}}-s\Theta -\frac{K}{s}=0$$

where $\Theta (\xi ;s)={\mathfrak{L}}_{\tau }[\Theta (\xi ,\tau )]$ denotes the Laplace transform of temperature and ${\Theta }^{{{\prime\prime}}}=\frac{{d}^{2}\Theta }{d{\xi }^{2}}$.

The general solution of Eq. (21) is

22 $$\Theta (\xi ;s)=A(s){e}^{\xi \sqrt{s}}+B(s){e}^{-\xi \sqrt{s}}-\frac{K}{{s}^{2}}$$

where $A(s)$ and $B(s)$ are arbitrary functions, to be found from satisfying the boundary conditions.

It is worth to remind here that the assumption of the domain’s being semi-infinite remains valid as long as the PCM can be treated as an infinite heat sink—the latter is true if the PCM operates in an optimal way, that is, near its phase-change temperature.

For a semi-infinite domain, $A(s)\equiv 0$, so that Eq. (22) reduces to

23 $$\Theta (\xi ;s)=B(s){e}^{-\xi \sqrt{s}}-\frac{K}{{s}^{2}}$$

The task now is not to solve Eq. (19) explicitly, but to establish its so-called non-field solution; namely, to derive an integral relation, which relates the local values of the temperature and the corresponding heat flux.

Differentiation of Eq. (23) with respect to $\xi$ yields

24 $$\frac{d\Theta (\xi ;s)}{d\xi }=-\sqrt{s}B(s){e}^{-\xi \sqrt{s}}$$

Summing up Eqs. (23) and (24) with rearranging the terms yields

25 $$\Theta =\frac{1}{\sqrt{s}}\left[-\frac{d\Theta }{d\xi }-\frac{K}{{s}^{3/2}}\right]$$

Taking the inverse Laplace transform of Eq. (25) and applying the convolution theorem results into

26 $$\theta (\xi ,\tau )=\frac{1}{\sqrt{\pi }}{\int }_{0}^{\tau }\left[-\frac{\partial \theta }{\partial \xi }-2K\sqrt{\zeta }\right]\frac{d\zeta }{\sqrt{\tau -\zeta }}$$

Upon restoring the original variables and rearranging the terms, the latter equation becomes

27 $$T(x,t)={T}_{0}+\frac{1}{\epsilon \sqrt{\pi }}{\int }_{0}^{t}\frac{{q}^{{{\prime\prime}}}(x,\zeta )d\zeta }{\sqrt{t-\zeta }}-K({T}_{f}-{T}_{0})$$

Equation (27) renders the desired non-field solution of Eq. (12).

Non-field solutions are known for that they relate local values of an intensive property—temperature in this case—and the corresponding flux. These solutions are valid everywhere within the domain including the boundaries. In view of the latter, non-field solutions proved to be extremely useful to transform boundary conditions of one kind into boundary conditions of another kind (e.g., the Dirichlet boundary conditions into the Neumann boundary conditions and vice versa)^11^.

In particular, the non-field solution, given by Eq. (27), can be inverted following the procedure adopted by Kulish and Lage^7^, that is,

28 $${q}^{{{\prime\prime}}}(x,t)=\epsilon \left[\frac{1}{\sqrt{\pi }}{\int }_{0}^{t}\frac{\partial T(x,\zeta )}{\partial \zeta }\frac{d\zeta }{\sqrt{t-\zeta }}+\frac{K({T}_{f}-{T}_{0})}{\sqrt{\pi t}}\right]$$

The non-field solutions, given by Eqs. (27) and (28), are used in the following section to analyse several practically important cases of the boundary conditions imposed upon THEDs with PCMs.

Furthermore, Eq. (27) allows one to introduce an expression of the effective thermal effusivity, which incorporated in itself the effect associated with the presence of the PCM. Indeed,

29 $$\frac{1}{{\epsilon }_{eff}\sqrt{\pi }}{\int }_{0}^{t}\frac{{q}^{{{\prime\prime}}}(\zeta )d\zeta }{\sqrt{t-\zeta }}=\frac{1}{\epsilon \sqrt{\pi }}{\int }_{0}^{t}\frac{{q}^{{{\prime\prime}}}(\zeta )d\zeta }{\sqrt{t-\zeta }}-K\left({T}_{f}-{T}_{0}\right).$$

After some algebra, the latter equation simplifies into

30 $$\frac{1}{{\epsilon }_{eff}}=\frac{1}{\epsilon }-\frac{K({T}_{f}-{T}_{0})\sqrt{\pi }}{{\int }_{0}^{t}\frac{{q}^{{{\prime\prime}}}(\zeta )d\zeta }{\sqrt{t-\zeta }}}$$

Notice that, in the case of a constant heat flux, Eq. (30) further simplifies into.

31 $$\frac{1}{{\epsilon }_{eff}}=\frac{1}{\epsilon }-\frac{K({T}_{f}-{T}_{0})}{2{q}^{{{\prime\prime}}}}\sqrt{\frac{\pi }{t}}$$

Notice also that, choosing ${q}^{{{\prime\prime}}}(t)={q}_{0}^{{{\prime\prime}}}/\sqrt{t}$, ${\int }_{0}^{t}\frac{{q}_{0}^{{{\prime\prime}}}d\zeta }{\sqrt{\zeta }\sqrt{t-\zeta }}=\pi {q}_{0}^{{{\prime\prime}}}$, so that Eq. (30) yields

32 $$\frac{1}{{\epsilon }_{eff}}=\frac{1}{\epsilon }-\frac{K\left({T}_{f}-{T}_{0}\right)}{{q}_{0}^{{{\prime\prime}}}\sqrt{\pi }},$$

where the second term in the right side is constant.

## Some practically important cases of the boundary conditions

One of the simplest and yet practically important cases of the boundary conditions to be imposed on TEHDs is the case of a constant heat flux through the TEHD’s boundary/surface, that is, ${q}_{s}^{{{\prime\prime}}}={q}_{0}^{{{\prime\prime}}}=const$. In this case, Eq. (27) renders a straightforward result for the temperature variation on the boundary, namely

33 $${T}_{s}(r,t)={T}_{0}+\frac{2{q}_{0}^{{{\prime\prime}}}}{\epsilon }\sqrt{\frac{t}{\pi }}-K({T}_{f}-{T}_{0})$$

In this case, however, the harvesting power of THEDs is constant with time and is fully defined by the geometry of the PCMs used, that is,

34 $${\stackrel{ }{Q}}_{hv}={q}_{0}^{{{\prime\prime}}}{A}_{PCM}$$

Geometrical constraints on the PCMs are considered in the following section.

If a TEHD is to operate in the course of diurnal cycles or other quasi-periodic regimes, the case of the periodic or quasi-periodic boundary conditions is to be considered. In the most general case, such a type of the boundary conditions is given in the form of the Fourier series

35 $${T}_{s}(t)=\stackrel{\infty }{\sum_{n=1}}\left[{a}_{n}cos({\omega }_{n}t)+{b}_{n}sin({\omega }_{n}t)\right]$$

where for the duration of a given cycle, $\Delta t$, ${\omega }_{n}=2\pi n/\Delta t$ and the corresponding Fourier coefficients are defined as follows

36a $${a}_{n}=\frac{2}{\Delta t}{\int }_{0}^{\Delta t}{T}_{s}(t)cos({\omega }_{n}t)dt$$

and

36b $${b}_{n}=\frac{2}{\Delta t}{\int }_{0}^{\Delta t}{T}_{s}\left(t\right)sin\left({\omega }_{n}t\right)dt.$$

For the boundary conditions in the form (35), Eq. (28) renders the value of the corresponding heat flux, that is,

37 $$\begin{array}{cc}{q}_{s}^{{{\prime\prime}}}(t)=& \epsilon \left\{\stackrel{\infty }{\sum_{n=1}}\sqrt{{\omega }_{n}}\left[{a}_{n}cos\left({\omega }_{n}t-\frac{\pi }{4}\right)+{b}_{n}sin\left({\omega }_{n}t-\frac{\pi }{4}\right)+\frac{K({T}_{f}-{T}_{0})}{\sqrt{\pi t}}\right]\right\}\\ +& \epsilon \sqrt{2}\stackrel{\infty }{\sum_{n=1}}\left[fres\left(\sqrt{\frac{2{\omega }_{n}t}{\pi }}\right)-gres\left(\sqrt{\frac{2{\omega }_{n}t}{\pi }}\right)\right]\end{array},$$

where the functions $fres()$ and $gres()$ are the auxiliary Fresnel integrals as defined by Abramowitz and Stegun^15^, namely:

38a $$fres(z)=\left[\frac{1}{2}-{\int }_{0}^{z}sin\left(\frac{\pi }{2}{\zeta }^{2}\right)d\zeta \right]cos\left(\frac{\pi }{2}{z}^{2}\right)-\left[\frac{1}{2}-{\int }_{0}^{z}cos\left(\frac{\pi }{2}{\zeta }^{2}\right)d\zeta \right]sin\left(\frac{\pi }{2}{z}^{2}\right)$$

and

38b $$gres(z)=\left[\frac{1}{2}-{\int }_{0}^{z}cos\left(\frac{\pi }{2}{\zeta }^{2}\right)d\zeta \right]cos\left(\frac{\pi }{2}{z}^{2}\right)+\left[\frac{1}{2}-{\int }_{0}^{z}sin\left(\frac{\pi }{2}{\zeta }^{2}\right)d\zeta \right]sin\left(\frac{\pi }{2}{z}^{2}\right)$$

**Figure 1:**
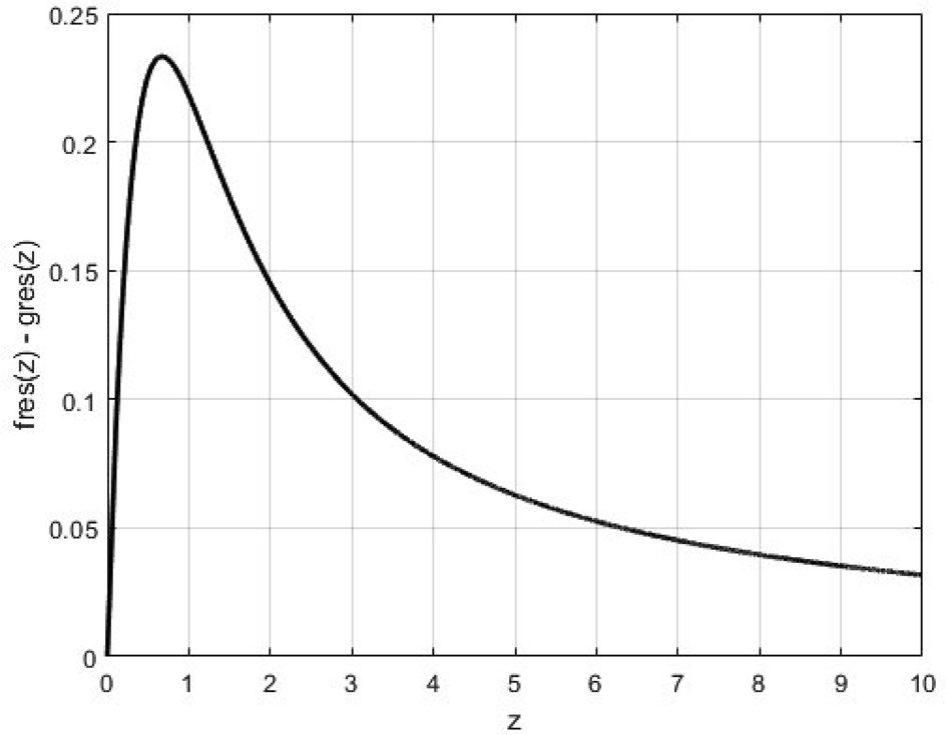
The value difference $fres(z)-gres(z)$ for $0\le z\le 10$.

Observe that, not only both the auxiliary Fresnel integrals converge to zero quite rapidly ($z=5$ for practical purposes), but also their values are of the same order of magnitude. In view of this, because the difference between the auxiliary Fresnel integrals defines the last term in Eq. (37), the contribution of these integrals can be neglected for most practical applications. To illustrate the statement made here, Fig. 1 shows the difference $fres(z)-gres(z)$.

Moreover, for practical purposes, it is sufficient to consider only major temperature variations during the operational cycle, such that

39 $${T}_{s}(t)={T}_{0}+(\Delta T{)}_{max}\left[\mathrm{cos}\left(\omega t\right)+\mathrm{sin}\left(\omega t\right)\right],$$

where $(\Delta T{)}_{max}$ is the maximal temperature variation in the course of a single operational cycle, the duration of which is $\Delta t$ and $\omega =2\pi /\Delta t$ is the characteristic frequency of the cycle.

The corresponding heat flux in this case is given by

40 $${q}_{s}^{{{\prime\prime}}}(t)=\epsilon \left\{(\Delta T{)}_{max}\sqrt{\omega }\left[cos\left(\omega t-\frac{\pi }{4}\right)+sin\left(\omega t-\frac{\pi }{4}\right)\right]+\frac{K({T}_{f}-{T}_{0})}{\sqrt{\pi t}}\right\}$$

The total amount of thermal energy, harvested during a single cycle, is then

41 $${Q}_{hv}={A}_{PCM}{\int }_{0}^{\Delta t}{q}_{s}^{{{\prime\prime}}}(\zeta )d\zeta =2\epsilon K\left({T}_{f}-{T}_{0}\right){A}_{PCM}\sqrt{\frac{\Delta t}{\pi }}$$

As can be seen from the latter equation, for a given duration of the harvesting cycle, the total amount of thermal energy, which can be harvested during that cycle, solely depends on the physical properties of the matrix material and PCM as well as the geometric configuration of the PCM. Hence, it becomes important to define geometrical constraints, which define the PCM performance.

## Geometrical constraints on the PCM

From Eq. (20) it follows that the PCM performance is directly proportional to the volume fraction of the PCM within the matrix material.

Consider a representative elementary volume (REV), which contains a single spherical PCM particle of diameter $d$—it is assumed here that the PCM particles are spherical. This particle is surrounded by the matrix material so that the total volume of the REV is

42 $$V=(d+\ell {)}^{3},$$

where $\ell$ is the average distance between the PCM particles within the matrix material. If the ratio $\ell /d=\beta$, then the volume fraction of the PCM is

43 $$\varphi =\frac{\pi }{6}{\left(\frac{1}{1+\beta }\right)}^{3}.$$

As follows from Eq. (43), the most compact packing of spherical PCM particles is achieved at $\beta =0$ yielding ${\varphi }_{max}=\pi /6\approx 0.5236$. However, such an arrangement does not meet either heat transfer or geometrical constraints.

As has been pointed out in “Optimal performance of thermal energy harvesting devices”, the optimal performance of the PCM is achieved if [see Eq. (6)]

44 $${t}_{hv}=\frac{{\ell }^{2}}{\alpha }.$$

Combining the latter equation with Eq. (7) yields the relation between $\ell$ and $d$, namely:

45 $$\ell =\frac{{\rho }_{PCM}{h}_{f}}{6\rho {c}_{p}\Delta T}d.$$

From the latter equation follows that the parameter $\beta$ to be used in Eq. (43), in order to guarantee the optimal performance of the PCM, must be

46 $$\beta =\frac{{\rho }_{PCM}{h}_{f}}{6\rho {c}_{p}\Delta T}$$

However, as have been demonstrated by Kulish and Lage^16^, if the PCM particles are located too close to each other, part of their surface area becomes shielded (blocked) by the neighbouring particles and, hence, excluded from the energy harvesting process. It has been found out that this happens when the volume fraction of the particles exceeds 16 per cent. Therefore, the maximal performance is achieved at *φ* = 0.16. The latter value has been determined from a numerical experiment with the relative error no greater than 0.1 per cent. At the same time, if *φ* < 0.16, part of the thermal energy, which could be stored within the PCM, would be conducted freely through the matrix material.

Hence, the most efficient thermal energy harvesting by the PCM is achieved at $\varphi =0.16$. Then, from Eq. (43), it follows that

47 $$\beta ={\left(\frac{\pi }{6\varphi }\right)}^{1/3}-1.$$

With $\varphi =0.16$, the latter equation yields $\beta \approx 0.485$, or $\ell \approx 0.485d$.

## General discussion

The main purpose of this concluding section is to formulate a concise procedure for the best choice of PCMs in TEHDs under a given set of the boundary conditions and geometrical constraints.

As pointed out in the preceding sections, the maximal amount of thermal energy is harvested if the entire surface area, which separates the PCM particles from the matrix material, is available for heat transfer. To achieve this, the volume fraction of the PCM is to be no more than 16 per cent. Then, it follows from Eq. (47) that the PCM particles are to be uniformly distributed within the matrix material, so that the distance between them equals almost one of their radius.

Equation (46) provides a guideline for the choice of the PCM material with respect to the physical properties of the matrix material, namely:

48 $$\frac{{\rho }_{PCM}{h}_{f}}{\rho {c}_{p}\left({T}_{f}-{T}_{0}\right)}=2.91,$$

where ${T}_{0}$ is a certain reference temperature (e.g., the ambient temperature).

Once the PCM material is chosen, the PCM performance can be quantified by Eq. (20), after which the heat transfer process within TEHDs with PCMs can be fully modelled by Eqs. (27) and (28), respectively. Obviously, an appropriate set of boundary conditions, under which the TEHD with PCMs operates, is to be imposed beforehand.

## Conclusions

In spite of significant growth of the body of knowledge about PCM performance and applications, some lack of understanding is still present^1–3^. In particular, no quantitative criteria are known for choosing PCM physical properties to provide their optimal performance.

In this study, for the first time: an expression for the characteristic harvesting time is derived under the optimal performance assumption from the constructal theory viewpoint and a dimensionless criterion, which characterises the performance of PCMs is introduced.

Furthermore, a non-field solution of the energy equation governing the heat transfer process within TEHDs with PCMs has been obtained. It is worth noting that non-field solutions were not used in previous studies for analysis of optimization problems. Hence, the material, presented in “Solution procedure” of this study, is of methodological importance—it extends the method of Kulish^10^ to solving optimization problems with energy sinks/sources. Using the non-field solution, a new expression for the effective thermal effusivity is then derived.

To illustrate the usefulness of the non-field solution, several important cases of the boundary conditions imposed on TEHDs with PCMs have been provided. In particular, the case of periodic or quasi-periodic boundary conditions is considered in more detail, because this case is important if energy harvesting is to be accomplished during diurnal cycles.

Finally, a new concise procedure for the best choice of PCMs in TEHDs under a given set of the boundary conditions and geometrical constraints has been formulated.

## Abbreviations

${c}_{p}$: Specific heat capacity, J/(kg K)
${h}_{f}$: Enthalpy of fusion, J/kg
$$k$$: Thermal conductivity, W/(m K)
$$m$$: Mass, kg
$Q$: Amount of heat, J
$${q}^{{{\prime\prime}}}$$: Heat flux, W/m^2^
${q}^{{{\prime\prime\prime}}}$: Volumetric heat sink, J/m^3^
$\dot{q}^{\prime\prime\prime}$: Volumetric heat dissipation, W/m^3^
$$r$$: Spatial variable, m
$s$: Laplace transform variable, 1/s
$$T$$: Temperature, K
$$t$$: Time, s
$$V$$: Volume, m^3^
$$z$$: Auxiliary variable

## Greek symbols

$$\alpha $$: Thermal diffusivity, m^2^/s
$$\epsilon $$: Thermal effusivity, J/(m^2^ K s^1/2^)
$\zeta$: Dummy integration variable, s
$\Theta$: Laplace transform of *θ*
$$\theta $$: Dimensionless temperature
$\xi$: Dimensionless spatial variable
$$\rho $$: Density, kg/m^3^
$$\tau $$: Dimensionless time
$$\varphi $$: Volume fraction

## Indices

0: Initial (equilibrium) state
cond: (Heat) conduction
f: Fusion
heat: Heating
hv: (Energy) harvesting
PCM: Phase-changing material
s: Surface (boundary)
th: Thermal (diffusion

## Special symbol

$\ell$: Characteristic length scale, m
